# Is surgical diagnostic excision always necessary for solid lesions with atypia?

**DOI:** 10.1186/bcr3761

**Published:** 2015-11-05

**Authors:** Nisha Sharma, Rebecca Millican-Slater, Eldo Verghese

**Affiliations:** 1 Leeds Teaching Hospital NHS Trust, Leeds, UK

## Introduction

As part of diagnostic work for radiological abnormalities seen in the breast, there has been an increase in use of vacuum-assisted biopsies for diagnosis. This allows more tissue to be sampled and therefore leads to a greater degree of diagnostic accuracy. In addition to diagnosis, in some centres the same procedure has also been used for removal of the entire lesion--vacuum-assisted excision (VAE). This is sometimes offered in place of a diagnostic surgical excision in cases of B3 lesions. We wanted to examine whether VAE can be a safe alternative for B3 lesion that show atypia.

## Methods

We identified all patients, at Leeds Teaching Hospital NHS Trust, who had undergone a surgical diagnostic excision following a core biopsy which had revealed the following lesions: fibroadenoma, papilloma or radial scar with atypia (FEA, AIDP or ISLN) during the period between 2009 and 2013. We reviewed the slides of the core biopsy and the subsequent excision biopsy to confirm the histological diagnosis.

## Results

Twenty-nine cases in total satisfied our inclusion criteria. There were nine cases of fibroadenomas with ISLN and/or AIDP. None of the cases showed upgrading of the atypia. There were eight cases of radial scar that had either ISLN, LCIS, epithelial atypia or AIDP, of which two showed DCIS in the surgical excision. There were 12 cases of papilloma with either ISLN or AIDP; of these, five had DCIS on surgical excision.

## Conclusion

VAE is safe for fibroadenomas with atypia and radial scars with atypia provided the periphery can be adequately sampled, to help diagnose DCIS. Papilloma with atypia requires surgical excision due to complex histological architecture.

